# Target vessel–specific efficacy and short-term outcomes of rotational atherectomy in the treatment of chronic total occlusion

**DOI:** 10.3389/fcvm.2025.1666668

**Published:** 2025-10-23

**Authors:** Bin Zhang, Yurong Sun, Hangrui Bai, Xiaojiao Zhang, Xinran Wang, Bo Luan

**Affiliations:** ^1^ Department of Cardiology, The People’s Hospital of Liaoning Province, The People’s Hospital of China Medical University, Shenyang, Liaoning, China; ^2^ Department of Cardiology, The Second Hospital of DaLian Medical University, Dalian, Liaoning, China; ^3^ Department of Cardiology, DaLian Medical University, DaLian, Liaoning, China

**Keywords:** chronic total occlusion, percutaneous coronary intervention, rotational atherectomy, short-term outcomes, target vessel

## Abstract

**Aim:**

To compare the procedural features, treatment efficacy, and short-term outcomes of RA for CTO in different target vessels—LAD, LCX, and RCA—and to assess the anatomical compatibility and clinical benefit of RA.

**Methods:**

This retrospective study included 119 patients with single-vessel CTO who underwent RA-assisted PCI from January 2019 to December 2024. Patients were divided into LAD (*n* = 55), LCX (*n* = 14), and RCA (*n* = 50) groups. Clinical characteristics, procedural details, and 6-month MACCEs were compared. Kaplan–Meier and Cox regression analyses were used to assess outcomes and predictors.

**Results:**

RCA-CTO patients were older and had more triple-vessel disease, but showed the lowest MACCEs rate at 6 months (10.0%) compared with LAD (23.6%) and LCX (28.6%) (*P* = 0.116). The difference between RCA and non-RCA groups was statistically significant (*P* = 0.042), the incidence was 10.0% in the RCA group and 24.6% in the non-RCA group (*post hoc*). Burr size and intravascular imaging use were similar across groups, though the RCA group had longer stents and used more contrast. Cox analysis showed potential trends between MACCEs and factors like imaging use, HDL, and triple-vessel disease, but none reached significance.

**Conclusion:**

RA-assisted PCI is safe and feasible for heavily calcified CTO lesions. Observed outcomes showed some variation by target vessel, with RCA-CTO appearing to have relatively better short-term results. Intravascular imaging may be helpful for guidance, but in this cohort it was not associated with a statistically significant reduction in 6-month MACCEs.

## 1 Introduction

Chronic total occlusion (CTO) represents one of the most technically challenging forms of coronary artery disease and is often regarded as “the last frontier” in interventional cardiology. In previous reports and studies (1–4), CTO lesions have been identified in approximately 20% of patients undergoing coronary arteriography (CAG). Moreover, CTO lesions are often accompanied by complex anatomical features, such as extensive calcification, severe fibrosis, long occlusion length, and poorly defined antegrade and retrograde channels, all of which pose significant challenges to percutaneous coronary intervention (PCI). Although recent advances in guidewire technology, microcatheter materials, retrograde techniques, and imaging-guided strategies have significantly improved the success rate of CTO-PCI (5), traditional balloon dilation strategies often prove inadequate in the presence of severely calcified and non-compliant lesions. This limitation increases the risk of procedural failure, periprocedural complications, and post-procedural restenosis.

Rotational atherectomy (RA) is a specialized technique for modifying calcified plaques by high-speed burr ablation, facilitating balloon dilation and stent delivery (6). While the use of RA in non-occlusive calcified lesions is well supported by extensive literature (7), its application in CTO, particularly in combination with retrograde recanalization strategies, remains insufficiently studied and lacks robust clinical evidence. RA is mainly considered in scenarios such as heavily calcified proximal caps, balloon-uncrossable or undilatable lesions, or failure of device delivery after guidewire passage (6). Current clinical research on RA in CTO primarily focuses on its overall feasibility and efficacy (8).

However, Anatomical and hemodynamic variations among vessels may influence burr advancement, guidewire manipulation, stent conformability, and restenosis risk. For example, RA in the Left Anterior Descending Artery (LAD) has been linked to higher short-term major adverse cardiovascular events (MACE) (9). Other studies have indicated that RA in the Right Coronary Artery (RCA) is more likely to result in chronic in-stent restenosis (ISR) (10). Moreover, RA is not a risk-free procedure and carries a higher complication risk, as complications such as dissection, perforation, slow/no-reflow, and side branch occlusion may occur (11). Therefore, systematically evaluating the impact of target vessel type on the technical success rate and short-term outcomes of RA for CTO lesions is crucial for optimizing RA application strategies in complex CTO cases.

## 2 Methods

### 2.1 Study population

This is a retrospective cohort study conducted at the people's hospital of Liaoning province between February 2019 and December 2024. The inclusion criteria were as follows: (1) age ≥ 18 years, regardless of gender; (2) Diagnosed CTO in one and only one major coronary artery [LAD, Left Circumflex Artery (LCX), or RCA], with RA utilized during the procedure; (3) Complete clinical data available and documented. Patients excluded were based on at least one of the following conditions: (1) Presence of CTO lesions in multiple coronary vessels; (2) History of intravenous thrombolysis or coronary artery bypass grafting (CABG); (3) In-stent total occlusion; (4) Acute myocardial infarction within the past month; (5) Patients with malignant tumors and an expected survival of less than one year; (6) Presence of other significant cardiac conditions, such as congenital heart disease, severe valvular heart disease, cardiomyopathy, serious arrhythmias, cardiogenic shock, or pulmonary heart disease. The study was reviewed and approved by the Ethics Committee of the People's Hospital of Liaoning province (Approval No.2024-K063).

### 2.2 Definitions

#### 2.2.1 CTO

CAG assessment showing complete interruption of antegrade flow (TIMI 0) or minimal contrast passage without effective distal vessel opacification (TIMI 1), with an estimated occlusion duration of ≥3 months (12).

#### 2.2.2 Procedural success

It was defined as successful recanalization of the target lesion confirmed by CAG, without the occurrence of any major adverse cardiovascular and cerebrovascular events (MACCEs) during the hospital stay. MACCEs included non-fatal myocardial infarction, cardiac death, non-fatal stroke, and target repeat revascularization (TVR), which encompassed repeat PCI or CABG.

#### 2.2.3 Current smoking

Defined as active smoking within the past three months, with a minimum of one cigarette per day.

#### 2.2.4 Diseased vessel

CAG confirmed ≥50% stenosis in a major coronary artery, with the number of diseased vessels primarily reflecting involvement of epicardial vessels.

#### 2.2.5 MACCEs

MACCEs were defined as non-fatal myocardial infarction, cardiac death, non-fatal stroke, and TVR. Non-fatal MI was defined according to clinical symptoms, elevated cardiac biomarkers, and/or electrocardiographic or imaging evidence of ischemia. Cardiac death included fatal MI, fatal stroke, sudden cardiac death, or other cardiac causes. Non-fatal stroke (ischemic or hemorrhagic) was diagnosed based on neurological deficits or imaging evidence. TVR was defined as any repeat intervention in the target or non-target vessel. Pericardial tamponade was counted only as a procedural event and was not included in MACCEs.

### 2.3 Data collection

All 119 enrolled cases included in this study were identified through outpatient and inpatient electronic medical record systems. Baseline clinical data were collected, including age, sex, body mass index (BMI), smoking history, family history of CAD, and relevant medical history. Blood samples (after at least 8 h of fasting) were obtained to measure routine blood parameters, liver and renal function, lipid profile, and blood glucose levels. All laboratory tests were performed by the Department of Laboratory Medicine, Liaoning Provincial People's Hospital.

Angiographic and PCI data were collected from operative and medical records, including the number of diseased vessels, number of CTO vessels, recanalization strategy (antegrade or retrograde approach), use of cutting balloons, use of intravascular lithotripsy (IVL), number of stents implanted, maximum stent diameter, total stent length, intraoperative use of intravascular imaging techniques(OCT or IVUS), and use of mechanical circulatory support devices(IABP, ECMO or Impella). Procedure-related complications occurring during hospitalization were also recorded. CTO target vessels were categorized according to the three main coronary arteries: LAD, LCX, and RCA.

### 2.4 Follow-up

Follow-up for all patients was conducted for 6 months, either through hospital visits or by telephone contact. Six-month follow-up was completed in 119/119 (100%). The primary endpoint of follow-up was the occurrence of MACCEs, consisting of cardiac death, non-fatal MI, TVR, and non-fatal stroke.

### 2.5 Statistical analysis

Patients were categorized into groups according to the three major coronary arteries: LAD, LCX, and RCA. Continuous variables were tested for normality. Data with a normal or approximately normal distribution were expressed as mean ± standard deviation (SD), and comparisons between groups were performed using the independent samples t-test. Continuous variables that did not conform to a normal distribution were expressed as median (25th–75th percentile), and intergroup comparisons were conducted using the rank-based nonparametric Kruskal–Wallis H test. Categorical variables were presented as frequencies and percentages. Intergroup comparisons were performed using the chi-square test (2 × 2) or Fisher's exact test (R × C), as appropriate. Primary comparison: LAD vs. LCX vs. RCA (two-sided *α* = 0.05). Planned pairwise tests used Bonferroni (*α* = 0.017). RCA vs. non-RCA was *post hoc* and is exploratory without further multiplicity adjustment; multivariable models were parsimonious and PH assumptions were checked. The Kaplan–Meier method was used to estimate survival curves, and the log-rank test was applied to evaluate the prognostic impact of RA in patients undergoing CTO-PCI. Cox proportional hazards regression models were employed to identify independent predictors of short-term outcomes. The results are reported as adjusted hazard ratios (HRs) along with the corresponding 95% confidence intervals (CIs). The entry and removal criteria for variables in the model were set at *p* = 0.05 and *p* = 0.10, respectively. All analysis was performed with SPSS 26.0. Figures were drawn via GraphPad Prism software (version 8.0.2).

## 3 Results

### 3.1 Characteristics of CTO patients

**Figure 1:**
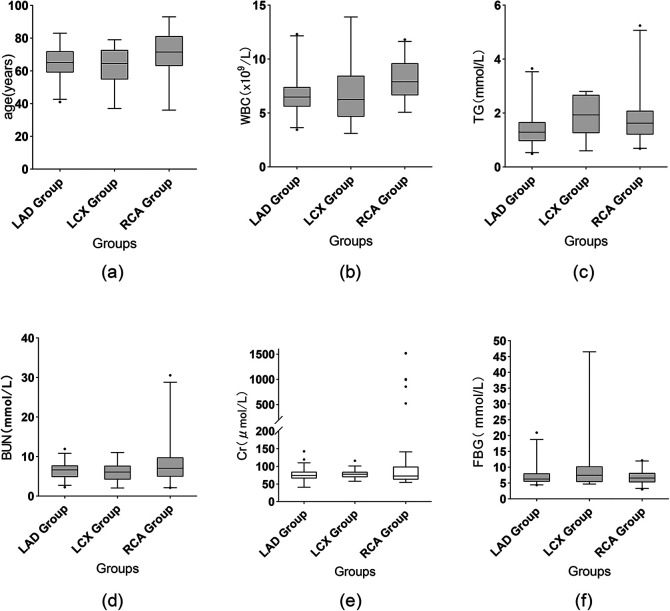
Comparison of selected data. Box: Interquartile range (IQR), spanning from the 25th to the 75th percentile. Horizontal line inside the box: Median (50th percentile). Whiskers (upper and lower lines extending from the box): Represent the 95% confidence interval. Small black points: Individual outliers beyond the caps. Creatinine is shown as box-and-whisker plots (whiskers = Q - 1.5×IQR to Q3 + 1.5×IQR, outliers as points).

A total of 119 patients were included in this study. Among them, 55 patients were in the LAD-CTO group, 14 in the LCX-CTO group, and 50 in the RCA-CTO group. Baseline characteristics are shown in Table 1. We report *p*-values for all variables; Bonferroni adjustment was applied only to *post-hoc* pairwise comparisons. As shown in Figure 1a, there was a significant difference in age among the three groups (*P* = 0.003). The mean age in the RCA-CTO group was 71.52 ± 12.21 years old, which was significantly higher than that in the LAD-CTO group (64.82 ± 9.76) and the LCX-CTO group (63.14 ± 11.67). In addition, although no statistically significant difference in sex distribution was observed among the three groups (*P* = 0.843), the overall population was predominantly male (68.9%), consistent with the epidemiological pattern that CTO is more commonly observed in men (13). Hypertension was present in 79 patients (66.4%), with significant differences among the three groups: 61.8% in the LAD-CTO group, 42.9% in the LCX-CTO group, and 78.0% in the RCA-CTO group (*P* = 0.003). There was no significant difference in the history of diabetes among the three groups (*P* = 0.540); however, the overall prevalence of diabetes was 58.0%, consistent with previous studies reporting a high prevalence of diabetes in patients with CTO (14). It is noteworthy that a significant difference was observed among the three groups in terms of prior PCI history (*P* = 0.029), with the highest proportion in the RCA-CTO group (72.0%) and the lowest in the LCX-CTO group (35.7%). This suggests that RCA-CTO lesions are more likely to be intervened upon or treated earlier, which may be related to differences in vascular anatomy, procedural complexity, and operator preference.

**Table 1:** Characteristics of CTO patients.

Variables	Overall (*n* = 119)	LAD-CTO group *n* = 55,46.2%	LCX-CTO group *n* = 14,11.8%	RCA-CTO group *n* = 50,42.0%	*P* value
Age	67.44 ± 11.53	64.82 ± 9.76	63.14 ± 11.67	71.52 ± 12.21^#^*	0.003
Male	82 (68.9%)	39 (70.9%)	10 (71.4%)	33 (66.0%)	0.843
BMI	24.83 ± 3.16	24.31 ± 2.75	25.02 ± 3.35	25.35 ± 3.48	0.233
Smoking	50 (42.0%)	24 (43.6%)	4 (28.6%)	22 (44.0%)	0.555
Family history of CAD	20 (16.8%)	7 (12.7%)	3 (21.4%)	10 (20.0%)	0.540
Hypertension	79 (66.4%)	34 (61.8%)	6 (42.9%)	39* (78.0%)	0.003
Diabetes mellitus	69 (58.0%)	29 (52.7%)	8 (57.1%)	32 (64.0%)	0.504
Prior Myocardial Infarction	60 (50.4%)	23 (41.8%)	7 (50%)	30 (60.0%)	0.177
Prior CVD	9 (7.6%)	5 (9.1%)	1 (7.1%)	3 (6.0%)	0.886
Prior PCI	71 (59.7%)	30 (54.5%)	5(35.7%)	36* (72.0%)	0.029

*P* < 0.05 indicates statistical significance. BMI, body mass index; CAD, coronary artery disease; CVD, cerebrovascular disease.

^#^ Denotes *P* < 0.017 compared with the LAD group;

* Denotes *P* < 0.017 compared with the LCX group.

### 3.2 Laboratory and diagnostic data

Some data demonstrated statistically significant differences among the different target vessels (Table 2). Firstly, WBC counts differed significantly among the three groups (*P* = 0.001), as shown in Figure 1b. The RCA-CTO group had the highest WBC count (8.07 ± 2.07 × 10⁹/L), while the LAD-CTO group had the lowest (6.71 ± 1.85 × 10⁹/L), suggesting a potentially more pronounced inflammatory state in patients with RCA occlusion. In terms of lipid metabolism, triglyceride (TG) levels showed a statistically significant difference among the three groups (*P* = 0.011), as shown in Figure 1c. The RCA-CTO group had a TG level of 1.84 ± 0.95 mmol/L, and the LCX-CTO group had a level of 1.89 ± 0.81 mmol/L, both of which were significantly higher than that of the LAD-CTO group (1.40 ± 0.64 mmol/L).

**Table 2:** Laboratory and diagnostic parameters.

Variables	Overall (*n* = 119)	LAD-CTO group *n* = 55,46.2%	LCX-CTO group *n* = 14,11.8%	RCA-CTO group *n* = 50,42.0%	*P* value
Hb (g/L)	132.16 ± 19.81	133.13 ± 17.70	135.79 ± 24.62	130.08 ± 20.73	0.566
RBC (10^12^/L)	4.28 ± 0.64	4.34 ± 0.63	4.30 ± 0.74	4.22 ± 0.63	0.660
WBC (10^9^/L)	7.28 ± 2.07	6.71 ± 1.85	6.67 ± 2.72	8.07 ± 2.07^#^*	0.001
PLT (10^9^/L)	215.72 ± 65.25	224.89 ± 66.27	206.64 ± 54.29	208.18 ± 66.75	0.366
ALT (U/L)	29.43 ± 27.56	29.84 ± 23.91	27.41 ± 16.19	29.55 ± 33.58	0.957
AST (U/L)	27.09 ± 16.04	28.24 ± 18.98	28.21 ± 16.02	25.52 ± 12.25	0.663
TC (mmol/L)	3.72 ± 0.97	3.65 ± 0.99	3.48 ± 0.72	3.87 ± 1.00	0.315
TG (mmol/L)	1.64 ± 0.83	1.40 ± 0.64	1.89 ± 0.81^#^	1.84 ± 0.95^#^	0.011
HDL (mmol/L)	1.00 ± 0.31	1.01 ± 0.32	1.04 ± 0.51	0.98 ± 0.23	0.775
LDL (mmol/L)	2.50 ± 0.81	2.49 ± 0.75	2.77 ± 1.13	2.43 ± 0.76	0.391
BUN (mmol/L)	7.15 ± 3.95	6.37 ± 1.87	6.15 ± 2.74	8.28 ± 5.42^#^	0.026
Cr (μmol/L)	74.0 (64.1,86.6)	74.0 (65.8,84.7)	77.5 (69.2,84.5)	72.6 (62.6,99.3)	0.777
UA (μmol/L)	407.37 ± 292.65	434.55 ± 417.72	378.43 ± 83.39	385.58 ± 102.75	0.645
FBG (mmol/L)	7.50 ± 4.53	7.32 ± 3.14*	10.61 ± 10.82	6.82 ± 2.02*	0.019
HbA1c (%)	6.62 ± 1.40	6.75 ± 1.71	6.51 ± 1.05	6.50 ± 1.09	0.637
LVEF (%)	47.01 ± 8.34	47.53 ± 8.48	48.64 ± 9.13	45.98 ± 7.99	0.473
Preprocedural imaging (*n*)					
CTA	60 (50.4%)	29 (52.7%)	9 (64.3%)	22 (44.0%)	0.365
CAG	75 (63.0%)	34 (61.8%)	5 (35.7%)	36* (72.0%)	0.044

*P* < 0.05 indicates statistical significance. Hb, hemoglobin; RBC, red blood cells; WBC, white blood cells; PLT, platelets; ALT, alanine aminotransferase; AST, aspartate aminotransferase; TC, total cholesterol; TG, triglycerides; HDL, high-density lipoprotein; LDL, low-density lipoprotein; BUN, blood urea nitrogen; Cr, creatinine; UA, uric acid; FBG, fasting blood glucose; HbA1c, glycated hemoglobin; LVEF, left ventricular ejection fraction.

^#^ Denotes *P* < 0.017 compared with the LAD group;

* Denotes *P* < 0.017 compared with the LCX group.

In addition, blood urea nitrogen (BUN) levels differed significantly among the three groups (*P* = 0.026), whereas creatinine (Cr) levels did not show a significant difference (*P* = 0.777), as shown in Figures 1d,e. The RCA-CTO group exhibited higher BUN levels (8.28 ± 5.42 mmol/L), while Cr levels were reported as median (IQR) [72.6 (62.6–99.3) μmol/L], without significant intergroup variation. Fasting blood glucose (FBG) levels also showed a statistically significant difference among the three groups (*P* = 0.019), as shown in Figure 1f. The LCX-CTO group had the highest FBG level (10.61 ± 10.82 mmol/L), indicating that patients with LCX occlusion may be more likely to have severe glucose metabolism disorders. Finally, in terms of preoperative imaging evaluation, the use of CAG showed a statistically significant difference among the groups (*P* = 0.044), with the highest utilization observed in the RCA-CTO group (72.0%). This may be related to the greater difficulty associated with recanalizing RCA occlusions.

### 3.3 CAG and PCI characteristics

Table 3 presents the procedural data of patients undergoing RA in different target vessel groups. Overall, no significant differences were observed among the three groups in most of the assessed parameters. Although there were no significant differences in J-CTO scores among the three groups (*P* = 0.857), the overall mean score was 3.00 ± 0.74, indicating a high procedural complexity for PCI in all patients. Additionally, there were no significant differences in burr size and rotational speed used among the three groups (*P* = 0.388 and *P* = 0.952). Regarding the number of diseased vessels, the majority in all three groups had triple-vessel disease, accounting for 41.8% in the LAD-CTO group, 64.3% in the LCX-CTO group, and 78.0% in the RCA-CTO group, with a statistically significant difference among the groups (*P* = 0.001). The RCA-CTO group also exhibited a significantly greater mean total stent length compared to the other two groups (RCA: 79.44 ± 33.33 mm; LAD: 65.29 ± 25.67 mm; LCX: 62.86 ± 36.42 mm), with the difference reaching statistical significance (*P* = 0.037). Furthermore, a significant difference was observed in contrast agent usage among the three groups (*P* = 0.002). The RCA-CTO group received the highest volume of contrast media (326.60 ± 72.10 ml), which was significantly greater than that used in the other two groups, consistent with findings from previous studies (15).

**Table 3:** Angiographic and procedural characteristics.

Variables	Overall (*n* = 119)	LAD-CTO group *n* = 55,46.2%	LCX-CTO group *n* = 14,11.8%	RCA-CTO group *n* = 50,42.0%	*P* value
Number of diseased coronary vessels					0.001
Single-vessel disease	15 (12.6%)	13 (23.6%)	1 (7.1%)	1 (2.0%)	
Double-vessel disease	33 (27.7%)	19 (34.5%)	4 (28.6%)	10 (20%)	
Triple-vessel disease	71 (59.7%)	23 (41.8%)	9 (64.3%)	39 (78.0%)	
CTO technique					0.564
Antegrade	88 (73.9%)	40 (72.7%)	12 (85.7%)	36 (72.0%)	
Retrograde	31 (26.1%)	15 (27.3%)	2 (14.3%)	14 (28.0%)	
J-CTO score	3.00 ± 0.74	2.98 ± 0.83	2.93 ± 0.62	3.04 ± 0.67	0.857
Cutting balloon	46 (38.7%)	20 (36.4%)	5 (35.7%)	21 (42.0%)	0.815
IVL	31 (26.1%)	16 (29.1%)	6 (42.9%)	9 (18.0%)	0.135
Number of stents implanted	2.29 ± 1.04	2.15 ± 0.95	2.57 ± 1.34	2.36 ± 1.05	0.319
Max diameter of implanted stent	3.16 ± 0.45	3.08 ± 0.44	3.13 ± 0.51	3.27 ± 0.44	0.107
Overall stent length (mm)	70.95 ± 31.02	65.29 ± 25.67	62.86 ± 36.42	79.44 ± 33.33^#^	0.037
Total procedural time (min)	117.49 ± 48.33	113.27 ± 51.34	130.07 ± 48.01	118.60 ± 45.31	0.502
Fluoroscopy time (min)	76.82 ± 38.10	73.89 ± 40.96	82.71 ± 35.26	78.38 ± 35.98	0.693
Max burr size used (mm)	1.47 ± 0.17	1.48 ± 0.14	1.51 ± 0.25	1.45 ± 0.17	0.388
burr rotational speed	14.84 ± 1.16	14.83 ± 0.96	14.93 ± 1.33	14.82 ± 1.34	0.952
Imaging Examination	112 (94.1%)	52 (94.5%)	14 (100%)	46 (92.0%)	0.752
Hemodynamic support	21 (17.6%)	10 (18.2%)	2 (14.3%)	9 (18.0%)	0.940
Contrast volume (ml)	292.10 ± 91.11	268.00 ± 79.41	263.57 ± 147.16	326.60 ± 72.10^#*^	0.002
Procedural success	111 (93.3%)	51 (92.7%)	13 (92.9%)	47 (94.0%)	0.965
In-hospital MACCEs	7(5.9%)	4(7.3%)	0	3(6.0%)	0.871

The mean J-CTO score of all CTO patients included in this study was 3.00 ± 0.74, indicating that the lesion complexity and difficulty of recanalization in all three groups reached a challenging level (16). Intravascular imaging techniques, including IVUS or OCT, were utilized in 94.1% of patients, which aligns with current interventional guidelines strongly recommending the use of intravascular imaging (17). During hospitalization, a total of 7 patients experienced procedure-related adverse events (Supplementary Table S1). In the LAD group, 4 events occurred, including one case each of vessel perforation, cardiac tamponade, urgent TVR, and acute cardiac decompensation. The RCA group had 3 events, including one case each of vessel perforation, cardiac tamponade, and acute cardiac decompensation. The overall incidence rate of 5.9% was lower than that reported in previous studies.

### 3.4 6 mouths follow-up for MACCEs

A total of 22 MACCEs events occurred during follow-up, with an overall incidence rate of 18.5% (Table 4). These included 2 cases of cardiac death, 11 non-fatal myocardial infarctions, 13 TVR, 4 non-fatal strokes, and 1 case of cardiac tamponade. Pericardial tamponade was counted only as a procedural event and was not included in MACCEs.

**Table 4:** Cumulative MACCEs at 6-month follow-up.

Variables	Overall (*n* = 119)	LAD-CTO group *n* = 55,46.2%	LCX-CTO group *n* = 14,11.8%	RCA-CTO group *n* = 50,42.0%	*P* value
MACCEs events	22 (18.5%)	13 (23.6%)	4 (28.6%)	5 (10.0%)	0.116
Cardiac mortality	2 (1.7%)	2 (3.6%)	0	0	0.608
Non-fatal myocardial infarction	11 (9.2%)	6 (10.9%)	3 (21.4%)	2 (4.0%)	0.090
TVR	13 (10.9%)	7 (12.7%)	3 (21.4%)	3 (6.0%)	0.221
Non-fatal stroke	4 (3.4%)	3 (5.5%)	0	1 (2.0%)	0.771
Cardiac tamponade	1 (0.8%)	1 (1.8%)	0	0	1.000

**Figure 2:**
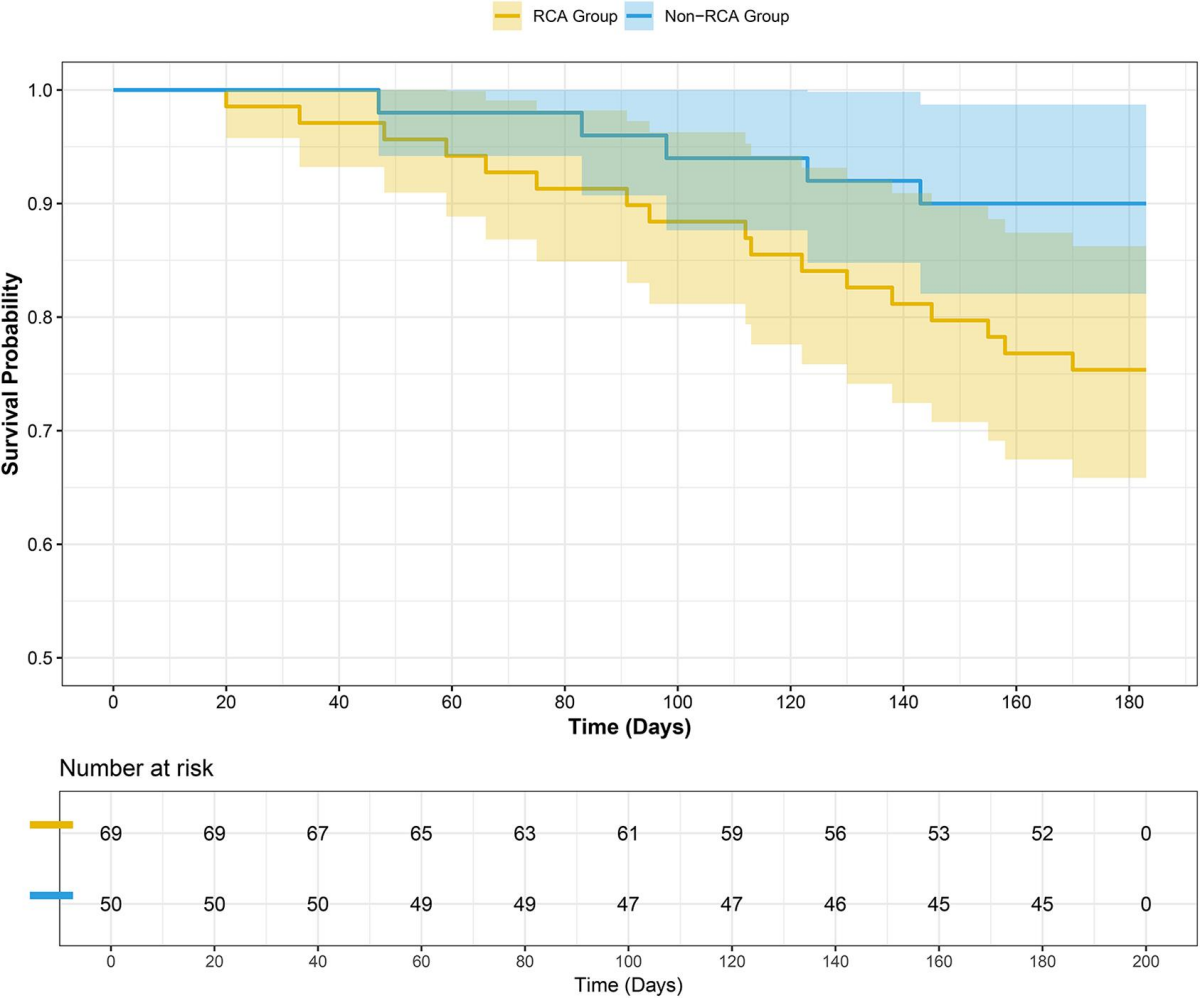
Survival curve. A significant difference was observed between the two groups (*P* = 0.046 Log-Rank test).

Comparison of MACCEs incidence among different CTO target vessels revealed no significant difference among the three groups (23.6% vs. 28.6% vs. 10.0%, *P* = 0.116). To further explore the impact of CTO lesion location on clinical outcomes, it was noted that there was no significant difference between the LAD and LCX groups (Chi-square partition test, *P* = 0.801). Meanwhile, comparisons between the RCA and LAD groups (*P* = 0.065) and between the RCA and LCX groups (*P* = 0.086) demonstrated a trend toward statistical significance. Therefore, for subsequent survival analysis, the LAD and LCX groups were combined into a non-RCA CTO group. As shown in Table 5, a significant difference was observed between the RCA-CTO and non-RCA CTO groups (*P* = 0.042). Survival curves were generated using the Kaplan–Meier method (Figure 2), and a significant difference was observed in MACCEs-free survival between the two groups (*P* = 0.046, log-rank test), indicating that patients in the RCA-CTO group had better short-term outcomes after RA. But the comparison between RCA and non-RCA was conducted *post hoc* and should be considered exploratory.

**Table 5:** Cumulative MACCEs at 6-month follow-up.

Variables	Overall (*n* = 119)	non-RCA-CTO Group *n* = 69,58.0%	RCA-CTO Group *n* = 50,42.0%	*P* Value
MACCEs events	22 (18.5%)	17 (24.6%)	5 (10.0%)	0.042
cardiac mortality	2 (1.7%)	2 (1.2%)	0	0.509
Non-fatal myocardial infarction	11 (9.2%)	9 (13.0%)	2 (4.0%)	0.174
TVR	13 (10.9%)	10 (14.5%)	3 (6.0%)	0.143
Non-fatal stroke	4 (3.4%)	3 (4.3%)	1 (2.0%)	0.638
Cardiac tamponade	1 (0.8%)	1 (1.4%)	0	1.000

### 3.5 Independent predictors of short-term outcomes

Covariates included in the analysis were age, sex, BMI, history of diabetes, history of hypertension, presence of triple-vessel disease, target vessel type (RCA vs. non-RCA), preoperative LVEF, Cr, BUN, hemoglobin, high-density lipoprotein (HDL), low-density lipoprotein (LDL), average number of stents implanted, total stent length, and use of intravascular imaging. A stepwise regression model was used for variable selection and analysis (Table 6). Cox regression included 16 covariates with 22 MACCE events; results should be interpreted as exploratory due to potential overfitting. Although differences in three-vessel disease, use of intravascular imaging, and HDL did not reach statistical significance (*P* = 0.079, *P* = 0.096, and *P* = 0.094, respectively), their observed directions are consistent with potential clinical relevance. However, given the limited sample size, these findings should be interpreted cautiously, and further studies with larger cohorts are needed to clarify their role in short-term prognosis.

**Table 6:** Cox regression analysis.

Variables	Overall (*n* = 119)					
	Univariate Cox			Multivariate Cox		
	HR	95%CI	*P* value	HR	95%CI	*P* value
Triple-vessel disease	0.352	0.148–0.839	0.019	0.435	0.172–1.100	0.079
Max burr size used	7.473	0.857–65.186	0.069	6.585	0.541–80.094	0.139
BUN	0.847	0.712–1.007	0.060	0.895	0.734–1.090	0.268
RCA-CTO	0.377	0.139–1.021	0.055	0.557	0.185–1.683	0.300
Imaging examination	0.308	0.091–1.043	0.058	0.335	0.092–1.215	0.096
HDL	4.003	1.513–10.592	0.005	2.360	0.863–6.451	0.094

## 4 Discussion

This study systematically investigated the application of RA in single-vessel CTO lesions, focusing on the differences in intraoperative characteristics, efficacy outcomes, and short-term prognosis of different target vessels. The findings revealed potential heterogeneity in the performance and impact of RA under varying anatomical conditions. This retrospective study applied strict inclusion and exclusion criteria, focusing on single-vessel CTOs and excluding in-stent occlusions or recent myocardial infarction, which may limit generalizability. A key limitation of this study is the absence of detailed lesion descriptors and procedural parameters, which may have introduced unmeasured confounding and selection bias related to unrecorded characteristics such as calcification, lesion length, and collateral circulation. We also acknowledge that Bonferroni correction is conservative and may miss some true differences. Khariton (18) suggested that recanalization of CTO lesions is beneficial for improving cardiac function in patients, regardless of baseline left ventricular function. Notably, despite the RCA-CTO group having older patients, a higher prevalence of hypertension, a higher proportion of triple-vessel disease, and more complex procedures (as reflected by greater contrast volume and longer total stent length), it exhibited the lowest MACCEs rate within 6 months. This may indicate that RA is particularly effective when used in the treatment of RCA-CTO lesions. From an anatomical perspective, CTO lesions are often morphologically associated with high-complexity features such as severe calcification, plaque eccentricity, and long lesion segments. These characteristics frequently render the lesions less responsive to conventional balloon dilation, and device delivery remains challenging even after successful guidewire crossing. As a technique of “selective ablation”, RA can effectively remove deep calcium deposits and improve vascular compliance, thereby creating a more favorable anatomical condition for stent deployment (19). Previous studies (20) have indicated that although RCA occlusions are often more complex, their anatomical characteristics—being distal to the left main artery (LM) and involving fewer bifurcation lesions compared to the left coronary artery(LCA)—generally offer a larger operative space and relatively more controllable procedural risk during RA. This may be one of the reasons why, in the present study, the RCA-CTO group exhibited better short-term outcomes despite having more complex lesions. Secondly, in terms of procedural approach and technical strategy, antegrade recanalization was preferred in all patients in this study. The rotational burr tends to advance along a relatively straighter and less resistant path within the RCA, facilitating smoother device delivery. In contrast, the LAD and LCX belong to the LCA system and are anatomically closer to the LM and its bifurcation region (21). Additionally, these two vessels have more extensive branching, resulting in a relatively confined space. During RA procedures, this necessitates more precise control of the burr's trajectory, as even slight deviations may lead to serious complications such as dissection, perforation, or side branch occlusion (22). Studies have reported (23) that the incidence of severe complications associated with rotational atherectomy ranges from 1.8% to 9.5%. Although the three groups in this study showed similar burr diameters, rotational speeds, and rates of intravascular imaging guidance, the RCA-CTO group demonstrated significantly better clinical outcomes. This suggests that RA may have higher compatibility and a more favorable risk-benefit profile when applied to RCA lesions. From the perspective of hemodynamics and microcirculation, the RCA primarily supplies the right heart system and the basal interventricular septum. Compared with the LAD, which provides critical blood flow to the anterior wall of the left ventricle, RCA occlusion may have a relatively smaller impact on global left ventricular function. Thus, when RA successfully recanalizes the RCA, the restored territory could represent more of a “functional border zone” rather than a region of high energy demand. This might partly contribute to less severe reperfusion-related effects and a more stable recovery, although this remains speculative. In addition, given the relative anatomical independence of the RCA, procedural failure in this vessel may be more manageable than in the left coronary system. These factors may help explain, at least in part, operators' greater willingness to employ RA in RCA lesions. From a pathological perspective, CTO lesions essentially represent highly mature atherosclerotic plaques. Their formation typically spans several months to years, during which chronic remodeling processes such as calcium salt deposition, collagen fiber cross-linking, and necrotic tissue encapsulation lead to characteristic features of dense calcification, reduced vascular compliance, and extensive fibrosis (24). Consequently, these lesions often respond poorly to conventional balloon dilation, representing a major cause of intraoperative device delivery failure and suboptimal stent deployment (25). However, it should also be noted that CTO lesions often result in prolonged myocardial hypoperfusion, leading to local metabolic disturbances that promote persistent infiltration of inflammatory cells such as macrophages and monocytes, thereby accelerating the formation of the necrotic core and the progression of calcification within the plaque (26). Although RA mechanically ablates plaques, the procedure may activate local inflammatory cascades. On one hand, the RA process can stimulate immune responses following endothelial injury, inducing the expression of inflammatory cytokines such as TNF-α and IL-6, which leads to transient endothelial activation and microcirculatory disturbances (27); On the other hand, if the burr during RA enters the lipid core or the calcified-necrotic transition zone, it may trigger plaque rupture, microthrombus release, or infiltration of inflammatory cells, thereby exacerbating the inflammatory response (28). Therefore, the ability of RA to achieve precise tissue ablation largely depends on intravascular imaging techniques and accurate control of the burr's trajectory.

A study has showed (29) that patients with renal dysfunction often exhibit more severe vascular calcification, leading to more complex lesions and poorer prognosis. However, in this study, although the RCA-CTO group had older patients, longer stent implantation, higher contrast media consumption, and worse renal function, the incidence of MACCEs was ultimately lower. Furthermore, as a retrospective study, key lesion characteristics (e.g., calcification severity, lesion length, and collateral circulation) were not systematically recorded, which may have introduced unmeasured confounding affecting the observed outcomes. This indicates that the effectiveness of RA depends not only on the complexity of the lesion itself but also, more importantly, on the degree of alignment among lesion anatomy, technical suitability, and operator strategy. Moreover, Kaplan–Meier survival analysis showed that patients with RCA-CTO had a significantly lower short-term incidence of MACCEs compared to the non-RCA-CTO group, further supporting the above hypothesis and aligning with findings from previous studies (30). In the Cox regression analysis, the use of intravascular imaging, presence of triple-vessel disease, and HDL levels did not reach statistical significance, but their observed directions were consistent with potential clinical relevance. These findings should be interpreted cautiously and warrant further investigation in larger studies to clarify their role in short-term outcomes. Some studies have indicated (31) that triple-vessel disease is one of the key factors influencing prognosis. Notably, the LCX-CTO group exhibited the highest incidence of MACCEs, which may be attributed to the inherent anatomical characteristics of the LCX, as well as suboptimal compatibility with RA techniques (30). However, this group included only 14 patients, limiting the statistical power of the analysis. Therefore, larger-scale studies are needed to confirm these findings.

From a mechanistic perspective, RA is not merely a tool for managing calcification; its broader role lies in reconstructing the compliance of the lesion segment and optimizing the local hemodynamic environment (32). Studies have shown that the restoration of local shear stress following RA may promote endothelial function recovery and inhibit neointimal hyperplasia (33), thereby reducing the risk of ISR (34). Furthermore, RA should be regarded as an integral component of a comprehensive interventional treatment strategy, rather than as an isolated technique. Within the traditional PCI framework, CTO recanalization is often regarded as the primary goal, and RA is treated merely as a technical tool to achieve it. However, the true value of RA lies not in whether the lesion can be opened, but in whether the reconstructed vessel possesses functional integrity, long-term stability, and a low risk of restenosis. Within this framework, differences in anatomical location, vessel wall structure, shear stress distribution, and metabolic characteristics of the perfusion territories among target vessels contribute to the asymmetry in functional restoration of the artery following RA. However, any incomplete stent deployment, inadequate endothelial healing, or abnormal shear stress may trigger neointimal hyperplasia (34), thereby increasing the risk of MACCEs. Therefore, the observed phenomenon in this study—that the RCA-CTO group exhibits better prognosis following RA treatment—does not simply reflect a higher technical success rate of RA in the RCA, but rather indicates that RCA-CTO lesions, once successfully reconstructed by RA, possess greater vascular integrity and functional recovery.

In addition to RA, IVL has recently emerged as a novel treatment modality for heavily calcified coronary lesions. IVL delivers pulsatile acoustic pressure waves that selectively fracture both superficial and deep calcium within the vessel wall while minimizing injury to the surrounding soft tissue. Clinical studies (35) have demonstrated its effectiveness in improving stent expansion and procedural safety in complex calcified lesions. Although longer-term data are still limited, IVL represents a promising complementary or alternative strategy to RA in selected patients (36), particularly when conventional techniques are insufficient to modify the lesion. Currently, RA is being explored in combination with other techniques such as IVL, cutting balloons, and drug-coated balloons to address different types of calcified lesions. In this study, the high utilization rate of RA combined with intravascular imaging highlights that, in highly complex lesions, the use of intravascular imaging significantly improves procedural outcomes and reduces postoperative adverse events (37, 38). This indicates that operators have begun to consciously optimize treatment strategies and refine procedural techniques, shifting from the traditional goal of merely overcoming device delivery challenges toward the concept of “targeted ablation and selective intervention” aimed at vascular function reconstruction. Such a paradigm shift holds promise for further enhancing the applicability and safety of RA in the treatment of CTO lesions.

## 5 Limitation

First, this study is a single-center retrospective analysis, which carries an inherent risk of selection bias, and the results may be influenced not only by the operator's technical expertise but also by individual procedural preferences, such as considering certain RCA-CTO anatomies more suitable for RA. Second, the sample size is relatively small, particularly in the LCX-CTO group, limiting the statistical power for between-group comparisons. Meanwhile, as this was a retrospective study, certain lesion-specific characteristics may have been subject to recording errors or missing data. Accordingly, factors such as calcification severity, lesion length, and collateral circulation were not systematically collected in the original dataset. This limitation may have introduced unmeasured confounding that could partially explain the observed differences in outcomes. Third, the follow-up period was limited to six months, preventing assessment of the long-term prognosis of RA across different target vessels. Future prospective multicenter studies with broader inclusion criteria, long-term follow-up, and comprehensive functional and imaging data will be required to validate and extend our findings, and to further investigate the differential application of RA across various CTO target vessels.

## 6 Conclusion

In highly calcified and non-compliant CTO lesions, RA-assisted treatment is generally safe and feasible, demonstrating relatively favorable safety and operability profiles. Differences in outcomes among RA-treated CTO lesions across target vessels may be related, in part, to anatomical characteristics such as those of the RCA, which could contribute to more favorable short-term prognosis. Intravascular imaging techniques may assist in guiding RA procedures by improving procedural precision and potentially reducing complications, although our study did not demonstrate a statistically significant outcome benefit.

## Data Availability

The raw data supporting the conclusions of this article will be made available by the authors, without undue reservation.
