# Supplementary material for: Target vessel–specific efficacy and short-term outcomes of rotational atherectomy in the treatment of chronic total occlusion
Source: Front Cardiovasc Med. 2025 Oct 23;12:1666668. doi: 10.3389/fcvm.2025.1666668 (PMC12590502; doi:10.3389/fcvm.2025.1666668)
Supplement: Supplementary file 1 [file Table1.pdf]

Supplementary Table 1 Cumulative In-hospital MACCEs

	Overall (n=119)	LAD-CTO Group n=55,46.2%	LCX-CTO Group n=14,11.8%	RCA-CTO Group n=50,42.0%
In-hospital MACCEs	7(5.9%)	4(7.3%)	0	3(6.0%)
coronary perforation	2(1.7%)	1(1.8%)	0	1(2.0%)
urgent TVR	1(0.8%)	1(1.8%)	0	0
acute heart failure decompensation	2(1.7%)	1(1.8%)	0	1(2.0%)
cardiac tamponade	2(1.7%)	1(1.8%)	0	1(2.0%)
